# LIGHT/TNFSF14 Affects Adipose Tissue Phenotype

**DOI:** 10.3390/ijms25020716

**Published:** 2024-01-05

**Authors:** Angela Oranger, Graziana Colaianni, Giuseppe Ingravallo, Vincenza Sara Scarcella, Maria Felicia Faienza, Maria Grano, Silvia Colucci, Giacomina Brunetti

**Affiliations:** 1 Department of Precision and Regenerative Medicine and Ionian Area, University of Bari, 70124 Bari, Italy; angela.oranger@uniba.it (A.O.); graziana.colaianni@uniba.it (G.C.); maria.grano@uniba.it (M.G.); 2 Section of Pathology, Department of Precision and Regenerative Medicine and Ionian Area (DiMePRe-J), University of Bari, 70124 Bari, Italy; giuseppe.ingravallo@uniba.it (G.I.); vincenza.scarcella@policlinico.ba.it (V.S.S.); 3 Pediatric Unit, Department of Precision and Regenerative Medicine and Ionian Area, University of Bari, 70124 Bari, Italy; 4 Department of Translational Biomedicine and Neuroscience, University of Bari, 70124 Bari, Italy; silviaconcetta.colucci@uniba.it; 5 Department of Biosciences, Biotechnologies and Environment, University of Bari, 70125 Bari, Italy

**Keywords:** LIGHT/TNFSF14, high fat diet, adipose tissue

## Abstract

LIGHT/TNFSF14 is linked to several signaling pathways as a crucial member of a larger immunoregulatory network. It is primarily expressed in inflammatory effector cells, and high levels of LIGHT have been reported in obesity. Thus, with the aim of deepening the knowledge of the role of LIGHT on adipose tissue phenotype, we studied wild-type (WT), *Tnfsf14^−^*^/*−*^, *Rag*^−/−^ and *Rag*-/*Tnfsf14*- (DKO) mice fed a normal diet (ND) or high-fat diet (HFD). Our results show that, although there is no significant weight gain between the mice with different genotypes, it is significant within each of them. We also detected an increase in visceral White Adipose Tissue (vWAT) weight in all mice fed HFD, together with the lowest levels of vWAT weight in *Tnfsf14*^−/−^ and DKO mice fed ND with respect to the other strain. Inguinal WAT (iWAT) weight is significantly affected by genotype and HFD. The least amount of iWAT was detected in DKO mice fed ND. Histological analysis of vWAT showed that both the genotype and the diet significantly affect the adipocyte area, whereas the number is affected only by the genotype. In iWAT, the genotype and the diet significantly affect mean adipocyte area and number; interestingly, the area with the least adipocyte was detected in DKO mice fed ND, suggesting a potential browning effect due to the simultaneous lack of mature lymphocytes and LIGHT. Consistently, Uncoupling Protein 1 (UCP1) staining of iWAT demonstrated that few positive brown adipocytes appeared in DKO mice. Furthermore, LIGHT deficiency is associated with greater levels of UCP1, highlighting the lack of its expression in *Rag*^−/−^ mice. Liver examination showed that all mice fed HFD had a steatotic liver, but it was particularly evident for DKO mice. In conclusion, our study demonstrates that the adipose tissue phenotype is affected by LIGHT levels but also much more by mature lymphocytes.

## 1. Introduction

LIGHT/TNFSF14 (homologous to Lymphotoxins exhibiting Inducible expression and competing with herpes simplex virus Glycoprotein D for herpes virus entry mediator (HVEM), a receptor expressed by T lymphocytes) is encoded by the *TNFSF14* gene, and it is linked to the activation of the NF-κB family of transcription regulators as a crucial member of a larger immunoregulatory network [1]. It is primarily expressed in inflammatory effector cells, such as natural killer cells, dendritic cells, neutrophils, macrophages, innate lymphoid cells, CD8 memory, and CD4 T cells, but not in regulatory T, B cells, or naïve T cells [2]. This particular pattern of expression suggests its involvement in acute inflammatory and adaptative immune responses. Its high expression in immune cells and inflamed tissues, as also demonstrated in coronavirus disease 2019 (COVID-19), led to the identification of LIGHT as a priority candidate for immunotherapy [3,4]. However, with the primary objective of extending the use of this immunotherapy to other diseases, different studies have explored the role of LIGHT in metabolic diseases, including obesity. Previously published papers have reported an increase in circulating LIGHT levels in obese adults as well as in children [5,6], Prader Willi syndrome subjects displaying genetic obesity [7,8], and mice subjected to High Fat Diet (HFD composition: 19 MJ kg^−1^, 35% of energy from carbohydrate, 42% from fat, 23% from protein) [9]. Conversely, an anti-inflammatory diet reduced systemic inflammation and thus LIGHT levels [10]. These findings prompted different researchers to study the effect of LIGHT on adipogenesis. In detail, in vitro, the treatment of human adipocytes with LIGHT led to the production of cytokines that are associated with inflammation, including interleukin (IL)-6, IL-8, and Monocyte Chemoattractant Protein-1 (MCP-1) [5]. However, interestingly, the same authors report that, although LIGHT increased the expression of its own receptor (herpesvirus entry mediator (HVEM), it also determined a reduction in the expression of transcription factors involved in adipogenesis, such as the peroxisome proliferator-activated receptor-gamma (PPARγ), together with the fatty acid synthase [5]. These results are also supported by Tiller’s findings [11], demonstrating that LIGHT inhibited lipid accumulation in vitro in different models of adipocytes. They hypothesized that LIGHT interfered with the early steps of adipogenesis with the decreased expression of PPARγ and CCAAT/enhancer-binding protein-α (C/EBPα) without affecting proinflammatory cytokine levels. Differently, Kim et al. reported that LIGHT/HVEM interaction contributes to adipose tissue inflammation via the increase in immune cell activation, which is associated with infiltration of peritoneal macrophages as well as CD4^+^ and CD8^+^ T cells [12]. However, as a contribution to the contrasting reported effect of the ligand LIGHT on adipogenesis, Liu et al. reported that in vitro mesenchymal stem cells treated with LIGHT differentiate in adipocytes [13]. Further studies reported that the LIGHT/Lymphotoxin beta receptor (LTβR) signaling pathway directly affected adipogenesis. In particular, LIGHT/LTβR interaction determines an NF-κB mediated inhibitor effect on adipocyte precursors, thus deviating their fate to lymphoid organ stromal cells [14], with consequent inhibition of adipogenesis in both brown and white fat [13,14]. This effect is associated with the inhibition of the expression of PPARγ and C/EBPα. Differently, Kou et al. [15,16], using the 3T3-L1 cell line, reported that LIGHT acts as an anti-beigeing factor inhibiting beige adipocyte development and also white adipogenesis. In HFD-fed *Tnfsf14*^−/−^ mice, the same authors demonstrated that subcutaneous WAT from these mice was enlarged with respect to WT mice. Thus, the authors suggested that LIGHT, as a cytokine produced by activated immune cells, supports immune cell activation at the expense of adiposity. Murine models of obesity using LIGHT deficient mice have not provided clear results [7,15,17]. In particular, in a model of HFD, *Tnfrsf14*^−/−^ mice developed increased obesity, glucose intolerance, and hepatosteatosis [9]. Interestingly, in ovariectomy, a model of adipose tissue inflammation, *Tnfsf14*^−/−^ mice displayed reduced fat mass and inflammation together with ameliorated glucose homeostasis [18]. Other authors analyzed the involvement of steroid hormones on adipose tissue inflammation, detecting differences according to gender, thus leading to a different accumulation pattern that is related to the different sex hormones [19]. It has been found that female mice are protected against HFD-induced metabolic changes while maintaining an anti-inflammatory environment in the intra-abdominal WAT with an increased T regulatory (Treg) cell population, whereas HFD-fed male mice display adipose tissue inflammation, hyperinsulinemia, and glucose intolerance [20]. Ovariectomy models demonstrated a role for estrogen in WAT inflammation, particularly by regulating the appearance of senescence-related T cells [21]. Other authors have demonstrated that ovariectomized mice fed HFD showed a proinflammatory phenotype of the adipose tissue with increased M1 macrophages as well as increased T cells, B cells, and NK cells with respect to mice with intact ovarian function. Consequently, the role of LIGHT in adipose tissue homeostasis/phenotype requires further investigation. Our work presented here focused on the role of lymphocytes in the activity of LIGHT in adipose tissue homeostasis.

## 2. Results

### 2.1. Effect of High-Fat Diet (HFD) on Wild-Type (WT), Tnfsf14^−/−^, Rag^−/−^ and Rag^−^/Tnfsf14^−^ (DKO) Mice

**Figure 1:**
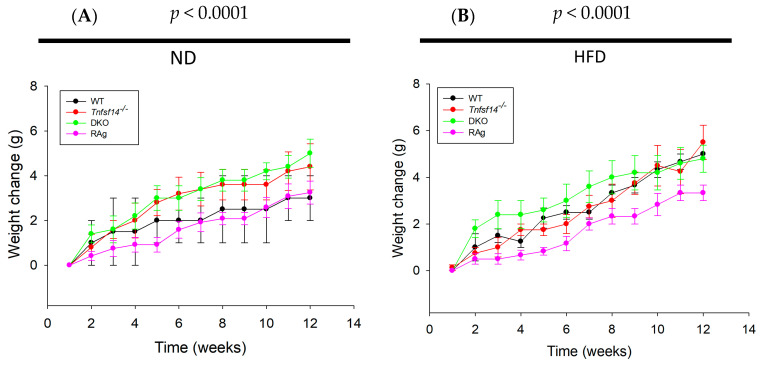
Weight change in mice fed with normal diet (ND, (**A**)) and high-fat diet (HFD, (**B**)). Significance was evaluated using repeated measurement analysis of variance (ANOVA) (*n* = 6).

**Figure 2:**
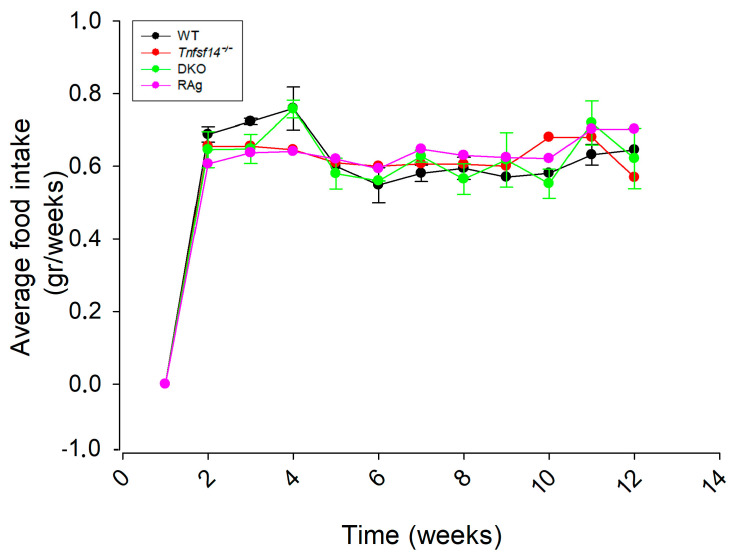
Average food intake of wild-type (WT), *Tnfsf14*^−/−^, *Rag*^−/−^, and *Rag^−^*/*Tnfsf14*^−^ (DKO) mice). Significance was evaluated using repeated measurement analysis of variance (ANOVA) (*n* = 6). All data were normalized to the total weight of the animals.

Fat homeostasis is regulated by M2 macrophages, neutrophils, eosinophils, dendritic cells, mast cells, innate lymphoid cells, and natural killer cells, B and T lymphocytes, with particular regard to Treg cells [21,22]. To deepen our knowledge of the role of LIGHT in fat homeostasis, we studied the effects of HFD on the fat phenotype of *Tnfsf14*^−/−^ compared with WT mice. The adaptive immune system has a crucial role in adipose tissue homeostasis; thus, the effect of HFD was also evaluated in *Rag*^−/−^, lacking mature T and B lymphocytes, as well as in DKO mice, an additional strain that simultaneously lacks both *Tnfsf14* and mature lymphocytes. The different mouse model fed with standard chow (ND) displayed significant differences in weight gain during the 12 weeks of treatment (*p* < 0.0001), but it was independent of the genotype, although the *Tnfsf14*^−/−^ and DKO mice displayed greater weight gain, suggesting the possible involvement of LIGHT (Figure 1A). Similarly, the mice fed the HFD also showed a significant increase in weight gain (*p* < 0.0001), which was genotype-independent. However, *Rag*^−/−^ showed the least weight gain, whereas DKO mice seemed to respond to the HFD with a rapid increase in weight change, but then it followed the same trend as WT and *Tnfsf14*^−/−^ mice (Figure 1B). During the experiments, we also evaluated the average food intake for all the different mouse models, but no mice registering significant differences were found (Figure 2).

**Figure 3:**
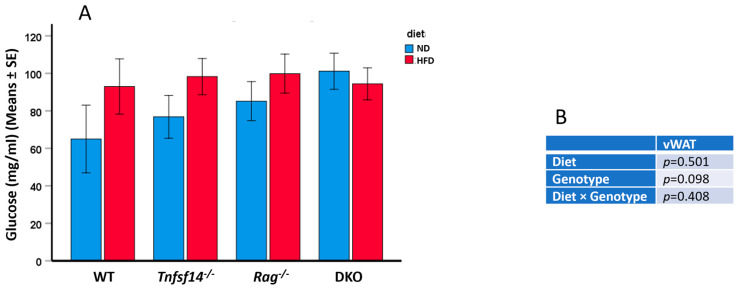
Blood glucose levels (mg/mL) of wild-type (WT), *Tnfsf14*^−/−^, *Rag*^−/−^ and *Rag^−^*/*Tnfsf14*^−^ (DKO) mice fed ND or HFD (**A**). The results of two-way ANOVA were reported in table (**B**), (*n* = 6).

The HFD affected the circulating glucose levels as well as the fat depots. In detail, WT, *Tnfsf14*^−/−^, and *Rag*^−/−^ fed HFD displayed a trend towards the statistically significant soft increase in glucose levels compared with the mice with the same genotype receiving an ND (*p* = 0.09) (Figure 3). Similarly, the different genotypes had no statistically significant effect on glucose levels (Table 1).

**Figure 4:**
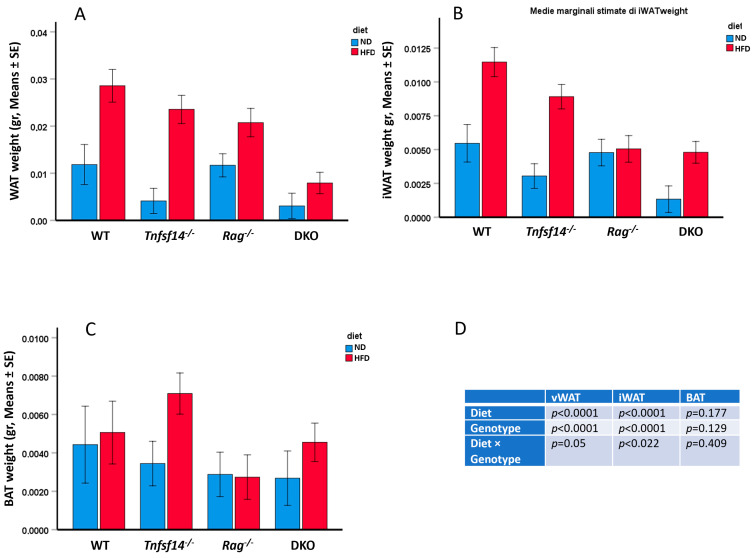
vWAT (**A**), iWAT (**B**), and BAT (**C**) weights of wild-type (WT), *Tnfsf14*^−/−^, *Rag*^−/−^ and *Rag^−^*/*Tnfsf14*^−^ (DKO) mice fed ND or HFD. The results of two-way ANOVA are also reported in table (**D**), (*n* = 6). All vWAT, iWAT, and BAT weights were normalized to the total weight of the animals.

Additionally, we measured the weights of visceral white adipose tissue (vWAT), inguinal WAT (iWAT), and brown adipose tissue (BAT) from mice fed ND and HFD diets (Figure 4, Table 1). Two-way ANOVA showed that vWAT weight was significantly affected both by the different genotypes and diets (Figure 4D) but not by their combination, although it was at the limit of significance (*p* = 0.05). The DKO mice showed the least amount of vWAT, and this difference was statistically significant compared to the WT, *Tnfsf14*^−/−^ and *Rag*^−/−^ of all other strains (*p* < 0.001, *p* = 0.021 and *p* = 0.019) (Figure 4A, Table 1). All mouse models fed the HFD compared with mice receiving the ND displayed a significant increase in vWAT weight (*p* = 0.005), Figure 4A.

The same analysis showed a significant effect of the genotype, diet, and their combination on iWAT weight (Figure 4B,D). As further detailed in Table 1, the genotype affects iWAT weight, which is consistently significantly different in WT mice with respect to that of *Tnfsf14*^−/−^*, Rag*^−/−^ and DKO mice (*p* < 0.001, for all), a significant difference also arises from the comparison of iWAT weight of *Tnfsf14*^−/−^ and DKO mice (*p* = 0.012). The combination of genotype and type of diet in iWAT weight gives significant results for the following conditions: a significant iWAT increase arose comparing WT and *Tnfsf14*^−/−^ mice fed ND with those fed HFD (*p* = 0.028 and *p* = 0.001), a significant difference was also evident for WT vs. *Rag*^−/−^ and DKO mice fed ND (*p* < 0.001, for all). Differently, for mice fed HFD, iWAT weight was significantly increased in WT mice compared to *Rag*^−/−^ and DKO mice (*p* = 0.002, *p* < 0.001, respectively).

Two-way ANOVA showed that BAT weight was not significantly affected by the different genotypes or diets, nor by their combination (Figure 4C,D, Table 1).

### 2.2. Histological Analysis of Adipose Tissues

**Figure 5:**
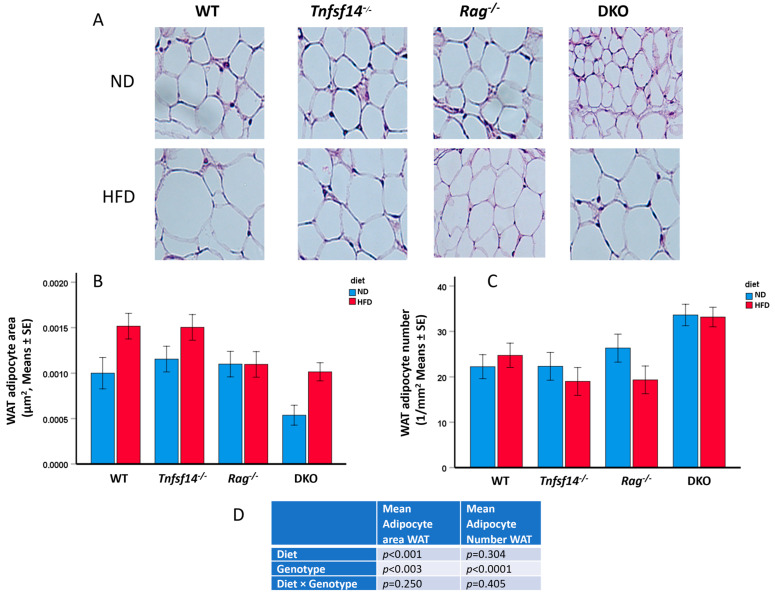
Microphotographs of vWAT of wild-type (WT), *Tnfsf14*^−/−^, *Rag*^−/−^ and *Rag^−^*/*Tnfsf14*^−^ (DKO) mice fed ND or HFD (**A**), together with the measurement of Mean Adipocyte Area (**B**), Mean Adipocyte number (**C**) and two-way ANOVA results in table (**D**), *n* = 6. Magnification 60×.

vWAT and iWAT from WT, *Tnfsf14*^−/−^, *Rag*^−/−^ and DKO mice fed ND or HFD were subjected to Hematoxylin/Eosin staining, and the evaluation of Mean Adipocyte Area and Mean Adipocyte number was performed. In detail, a different morphology of adipocytes can be observed from the histological staining of vWAT, showing a greater area of the cells in mice fed HFD compared with those fed ND (Figure 5A). Consistently, two-way ANOVA results showed that the mean adipocyte area was significantly affected by the type of diet as well as the genotype but not by their combination (Figure 5B–D). In detail, the area is significantly different when comparing DKO mice with WT and *Tnfsf14*^−/−^ mice (*p* = 0.009 and *p* = 0.001). The type of diet always affects the mean adipocyte area (*p* = 0.003, Table 1). From the interaction of these two parameters, significant results arise from the comparison of the mean adipocyte area of DKO mice fed ND with respect to WT and *Tnfsf14*^−/−^ fed HFD or *Tnfsf14*^−/−^ fed ND (*p* < 0.001, *p* < 0.001 and *p* = 0.042, Table 1). The evaluation of the adipocyte number using two-way ANOVA showed that only the genotype significantly affected this parameter, but not the type of diet or their interaction (Figure 5C,D). As detailed in Table 1, significant differences emerged following the comparison of DKO with WT, *Tnfsf14*^−/−^, and *Rag*^−/−^ mice (*p* = 0.003, *p* < 0.001, and *p* = 0.004, respectively), as in DKO mice, it was possible to count the highest number of adipocytes.

**Figure 6:**
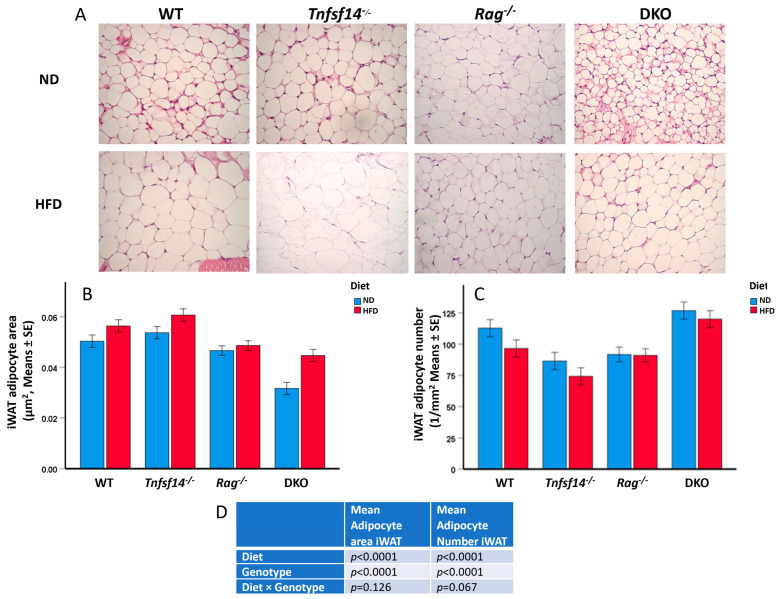
Microphotographs of iWAT of wild-type (WT), *Tnfsf14*^−/−^*, Rag*^−/−^ and *Rag*^−^/*Tnfsf14*^−^ (DKO) mice fed ND or HFD (**A**), magnification 40×, together with the measurement of Mean Adipocyte Area (**B**) and Mean Adipocyte number (**C**), as well as two-way ANOVA results in table (**D**), *n* = 6).

**Figure 7:**
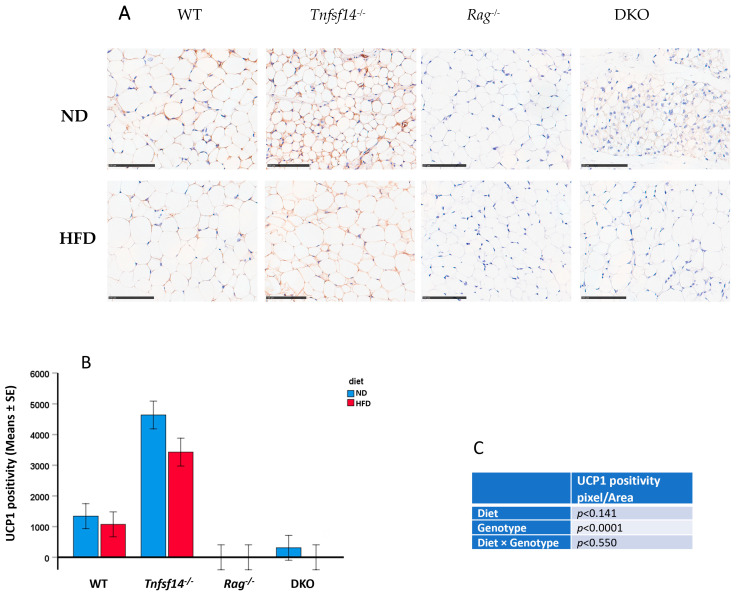
UCP1 immunohistochemical staining of iWAT sample from wild-type (WT), *Tnfsf14*^−/−^, *Rag*^−/−^ and *Rag*^−^/*Tnfsf14*^−^ (DKO) mice fed ND or HFD (**A**), together with the measurement of the positive area of staining (**B**) with the results of two-way ANOVA in table (**C**), *n* = 6. Bars indicate 100 µm.

As for iWAT, a different adipocyte morphology can be observed from the histological staining, showing a greater area of the cells of WT, *Tnfsf14*^−/−^*, Rag*^−/−^ and DKO mice fed HFD compared with those fed ND (Figure 6A). In detail, two-way ANOVA showed that the increase in iWAT adipocyte area was significantly linked to the genotype and the different diet but not to the interaction between these two parameters (Figure 6B, Table 1). The least adipocyte area was measurable in DKO mice compared with WT, *Tnfsf14*^−/−^*,* and *Rag*^−/−^ mice (*p* < 0.001, *p* < 0.001, and *p* = 0.02, respectively), the comparison between *Tnfsf14*^−/−^*,* and *Rag*^−/−^ was also associated with significant results (*p* = 0.002). The diet was significantly associated with the increase in iWAT adipocyte area (*p* = 0.002). Only some combinations of genotypes and diet resulted in a significant increase: DKO fed an ND with respect to the same mice fed HFD (*p* = 0.021), as well as DKO fed an ND compared with WT, *Tnfsf14*^−/−^*, Rag*^−/−^ (*p* < 0.001, *p* < 0.001, and *p* = 0.002). For mice following the HFD diet, the following significant differences were detected: WT vs. DKO (*p*= 0.049), *Tnf14*^−/−^ *vs. Rag*^−/−^ (*p* = 0.016) and DKO (*p* = 0.003). Consistently with the described results, the mean adipocyte number in iWAT was significantly affected by the genotype and the diet, with a trend toward significance for the interaction between these parameters (Figure 6C,D). The reduction observed comparing WT to *Tnfsf14*^−/−^ mice (*p* = 0.010) was significant for the genotype, with higher significant numbers measured for DKO mice compared with *Tnfsf14*^−/−^ and *Rag*^−/−^ (*p* < 0.001 for both). Some interactions between genotype and the type of diet were statistically significant: among mice fed ND, the iWAT adipocyte number was higher for DKO mice compared to *Tnfsf14*^−/−^ and *Rag*^−/−^ (*p* = 0.01 and *p* = 0.018), whereas for mice fed HFD the increase in DKO mice was significant compared to that in *Tnfsf14*^−/−^ mice (*p* = 0.003). The significant reduction in adipocyte number, together with the increase in number suggested a possible browning effect that we evaluated using immunostaining for UCP1, a marker of brown adipocytes (Figure 7A). Interestingly, we found that the expression of UCP1 is strictly linked to the genotype, as supported using two-way ANOVA. *Tnfsf14*^−/−^ mice displayed the highest positivity (*p* < 0.001 vs. WT, *Rag*^−/−^ and DKO mice), which was also retained following the HFD diet; *Rag*^−/−^ mice were negative for UCP1 expression. HFD diets reduced UCP1 positivity in WT, *Tnfsf14*^−/−^ and DKO mice. Small areas of UCP1 positivity were detected in DKO mice. However, in this last condition, the phenotype of adipocytes was different; they appeared as multilocular adipocytes. Looking at the interaction between genotype and diet, there was an evident statistical significance between *Tnfsf14*^−/−^ mice fed ND compared with those fed HFD (*p* = 0.011); for mice fed ND, the decrease occurring between *Tnfsf14*^−/−^ versus *Rag*^−/−^ and DKO mice was statistically significant (*p* < 0.001 for all the conditions), the same strain maintained the significance following the HFD diet (*Tnfsf14*^−/−^ versus *Rag*^−/−^ and DKO *p* < 0.001, respectively).

### 2.3. Histological Analysis of Liver

**Figure 8:**
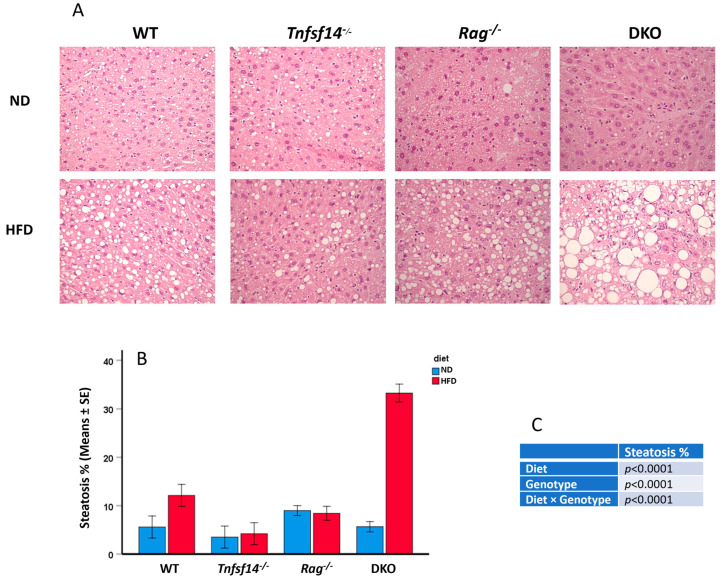
Microphotographs for the histological evaluation of liver of wild-type (WT), *Tnfsf14*^−/−^, *Rag*^−/−^ and *Rag*^−^/*Tnfsf14*^−^ (DKO) mice fed ND or HFD (**A**), evaluation of steatosis by determining the area of lipid in the sections (**B**) and results of two-way ANOVA (**C**). Magnification 40×.

Steatosis in livers from WT, *Tnfsf14*^−/−^, *Rag*^−/−^, and DKO mice fed ND or HFD were evaluated using microscopy (Hematoxylin/Eosin staining) (Figure 8). All mice fed HFD displayed a steatotic liver, but it was particularly evident for DKO mice, in which it seems that the vacuoles displaced the nucleus to the side nuclei; thus, it is possible to define macrovesicular steatosis [23]. Two-way ANOVA results showed that the genotype, diet, and interaction between these parameters all significantly affect this phenotype (Figure 8C). In detail, the highest increase associated with DKO mice was statistically significant with respect to all the other genotypes (*p* < 0.001, Table 1). The diet always significantly affected liver steatosis (*p* < 0.001, Table 1). The significance arising from the comparison of DKO mice fed ND vs. HFD diet (*p* < 0.001), in which the highest steatosis percentage developed, was particularly evident. This elevated increase was significant compared to all the conditions (Table 1). The obtained results suggest that the simultaneous deficiency of LIGHT and lymphocytes strongly participate in maintaining liver homeostasis.

## 3. Discussion

We have demonstrated the crucial role of LIGHT as well as T and B lymphocytes in determining the fat phenotype in mice, changing the amounts of vWAT and iWAT, but not BAT. We used mice deficient in *Tnfsf14* and *Rag*, or both genes, to determine their role in response to a high-fat diet.

The interest in exploring the role of LIGHT in HFD arises from the high levels of this cytokine detected in obesity, together with the conflicting results on the role of LIGHT in adipogenesis. The increased circulating levels of LIGHT have been demonstrated in obese pediatric and adult subjects, but also in genetic obesity associated with Prader-Willy patients [6,7,8]. Consistently, different murine models were used to study the role of LIGHT in obesity [13,14,15,16,17,18,24], and the complexity is also associated with the heterogeneous microenvironment that characterizes fat tissue. In fact, this tissue includes not only adipocytes but also immune cells that can present a different phenotype according to the inflamed status as well as the type of diet. Our results represent an advance with respect to the previous study as we have tried to evaluate the role of LIGHT on fat tissue homeostasis, also exploring the role of immune cells using *Rag*^−/−^ and DKO mice. *Rag*^−/−^ mice lack mature B and T lymphocytes, which allows the information arising from DKO mice to simultaneously lack lymphocytes and LIGHT protein expression. The use of this model can be useful to overcome the different results associated with in vitro and in vivo findings. In vitro, Liu et al. reported that LIGHT induces adipogenesis in mesenchymal stem cell cultures using LTβR [13], whereas Bassol et al. demonstrated that LIGHT treatment of mature adipocytes led to the production of proinflammatory cytokines via the HVEM receptor via NF-κB activation [5].

In vivo, different authors have explored the role of LIGHT in HFD-fed mice. In detail, Saunders et al. used 8-week-old mice and fed them with an ND or HFD (42% fat) for 12 weeks [9]. They demonstrated an increase in weight change only in HFD-fed *Tnfsf14*^−/−^ mice, which is also associated with adipocyte hypertrophy and liver steatosis, possibly due to an increase in hepatic lipase and IL-10 together with a decrease in IL-6. These reported data are in line with our results showing the increase in adipocyte area in WAT but differed from weight gain, which may be due to the younger mice used by Saunders et al. compared with animals in our study (8- vs. 12-week-old, respectively). Kou et al. [15] reported that LIGHT acts as an anti-beigeing factor by treating 6–8 week-old male WT and *Tnfsf14*^−/−^ mice for approximately 20 weeks with ND or HFD (60% of calories from fat), thus demonstrating a greater weight gain in HFD-fed *Tnfsf14*^−/−^ mice, which was not associated with significant variations in average food intake. Differently from our results, the detection of the significant weight gain may be due to the use of younger mice as well as to the prolonged HFD feeding (20 weeks by Kou vs. 12 weeks reported here). The results of Kuo et al. depicting LIGHT as an anti-beigeing cytokine [15] are consistent with our results showing the lowest area of adipocytes in iWAT from DKO mice that is associated with the detection of a small area of brown adipocytes, together with the greater expression of UCP1 in *Tnfsf14*^−/−^ compared to all strains supported. This anti-beigeing effect of LIGHT is more evident in the absence of T and B lymphocytes.

These results also support our previous findings showing a reduced bone mass of *Tnfsf14*^−/−^ mice compared to WT mice as well as the increased bone mass in DKO mice compared to *Rag*^−/−^ [25]; in fact, beige/brown adipose tissue exerts an anabolic effect on bone. BAT is a source of cytokines, such as irisin, leptin, fibroblast growth factor 21 (FGF21), IL-6, Wnt10b, and neuregulin 4, all of which could influence bone remodeling [26,27,28,29,30]. Irisin is a myokine with an anabolic effect on healthy and diseased bone [26,27,28]. Leptin, a well-studied protein, has a direct anabolic effect on osteoblasts, and BMSCs rich in leptin receptor (LepR) are the main source of bone formation [29,30]. Studies in healthy adults found a positive association between plasma FGF21 levels and BMD in women; furthermore, FGF21 is negatively related to regional BMD in humans; inconsistent results have also been reported in animal studies [29,30].

Herrero-Cervera et al. [17] fed WT and *Tnfsf14*^−/−^ mice with ND or a High Fat High Cholesterol diet (HFHCD—10.8% fat, 0.75% cholesterol), starting at the 8 week-age and followed for 16 weeks. The authors demonstrated that *Tnfsf14*^−/−^ mice fed this HFHCD diet displayed improved glucose tolerance and reduced hepatic inflammation and non-alcoholic fatty liver (NAFL). *Tnfsf14*^−/−^ displayed increased anti-inflammatory F4/80^+^CD206^+^ adipose tissue macrophages together with decreased F4/80^+^11c^+^ adipose tissue macrophages, thus supporting the idea that adipose tissue regulates liver homeostasis. The same authors also detected a reduced systemic inflammation with decreased circulating levels of Tumon Necrosis Factor (TNF) and IL-6, as well as adipose tissue cytokine secretion (MCP1, TNF, and IL-17). Our data demonstrate a trend towards the increase in glucose levels in mice fed HFD compared with mice fed ND in all models, except for DKO mice that already showed greater glucose levels in basal conditions. Our study also evaluated the fat accumulation in the liver, detecting the highest increase following HFD only in DKO mice, supporting the key role of LIGHT through T and B lymphocytes in liver homeostasis. However, it is important to underline that in DKO mice, the increase is very high, with a complete change in liver morphology and a high percentage of steatosis, suggesting that both LIGHT and mature lymphocytes prevent fat accumulation in the liver. These results are supported using literature data demonstrating that *Rag*^−/−^ mice following an HCHFD diet displayed greater hepatic inflammation via the increases in proinflammatory M1-like macrophages, natural killer cells, and granulocyte numbers, although they lack mature lymphocytes [31]. This situation, associated with other compensatory mechanisms due to LIGHT deficiency, could be responsible for the exacerbation of fat accumulation in the liver. Although it is important to underline that LIGHT deficiency is associated with reduced hepatic inflammation [17], and consistently, we did not find an increase in the steatosis percentage in these mice.

Interestingly, literature data also report the effect of HFD on *Rag*^−/−^ mice [24]. In 3-week-old male *Rag*^−/−^ mice fed a low-fat diet (LFD) or HFD for 28 weeks. Consistent with our results, the authors did not find a significant difference in weight gain nor detect any important differences in glucose levels. By comparing LFD to HFD-fed mice, they also identified a trend towards an increase in hepatic steatosis together with a significant increase in the adipocyte area, which is consistent with our results. Interestingly, in the Maugham et al. study [24], *Rag*^−/−^ fed LFD did not display white adipose tissue depots; however, it increased following HFD. Furthermore, we also demonstrate that adipocytes from iWAT of ND and HFD *Rag*^−/−^ mice did not express UCP1, suggesting a possible impairment of beigeing in mice lacking T and B lymphocytes, which represent the major source of LIGHT.

It is important to underline that all the described morphological changes of vWAT and iWAT are associated with different weight changes according to the different strains without significant changes in the average food intake.

## 4. Materials and Methods

### 4.1. Mice, Food Composition and Study Design

C57bl/6 mice deficient in *LIGHT* (*Tnfsf14*^−/−^*)* were obtained as previously described and were backcrossed (*n*  =  10). *Rag*^−^/*Tnfsf14*^−^ double knockout (DKO) mice were generated by crossing a *Rag*^−/−^ homozygous mouse with a *Tnfsf14*^−/−^ homozygous mouse. Subsequent generations of heterozygotes were bred together to produce a line with homozygous knockouts of both the *Rag* and *Tnfsf14* genes. Tnfsf14 heterozygous and *Rag-*/*Tnfsf14*^−^ mice were kindly provided by Prof Carl F Ware. *Rag*^−/−^ mice were purchased from Jackson Laboratory (Bar Harbor, ME, USA) and acclimatized to their housing for 3 weeks prior to starting the experiments. Forty-eight male animals were housed 4 per cage (to avoid stress caused by single housing) at 23 °C on a 12 h light/dark cycle and were fed a standard rodent chow for 3 months. Six mice for each genotype were randomly subjected to ND or HFD for an additional 3 months. Mice had free access to water and chow. Body weight was evaluated weekly, together with the food intake. At the end of the experimental procedure, overnight fasted mice were weighed and euthanized, and then their tissues were surgically excised and weighed. At sacrifice, glucose levels were measured in whole blood using a commercially available glucometer (PIC from Artsana, Grandate, Italy).

**HFD composition:** Lard, casein powder, maltodextrin, sucrose, soybean oil, calcium carbonate, monopotassium phosphate, sodium chloride, mineral dicalcium phosphate, potassium sulfate, magnesium oxide, nutritional additives (Vitamin A, Vitamin D3, Ferrum, Manganese, Zinc, Copper, Iodium, Selenium, Molibdenum), preservative additive (potassium citrate), colorant (blue indigotine). Analytical constituents (% by weight: crude proteins 23.00%, crude oils and fats 34.00%, crude fibers 5.00%, and crude ash 5.50% (Envigo, Madison, WI, USA); (% Kcal from Protein 18.3%, Carbohydrate 21.4%, Fat 60.3%).

### 4.2. Adipose Tissue Histology

At sacrifice, different adipose tissue depots, including visceral white adipose tissue (vWAT), inguinal WAT (iWAT), and interscapular brown adipose tissue (BAT), were collected and weighed. Then, vWAT, iWAT, and BAT were fixed with 4% (vol/vol) paraformaldehyde/PBS for 24 h at 4 °C, embedded in paraffin, sectioned, and stained with hematoxylin/eosin (H&E). The Mean adipocyte area was evaluated in sections of vWAT, iWAT, and BAT using Image J software (version 1.52, realized by National Institutes of Health, USA). Moreover, iWAT sections were processed for staining for UCP-1 (1:2000, Abcam, Cambridge, MA, USA). The reaction was revealed with the Dako EnVisionTM FLEX+ detection system (Dako Italia S.p.A., Milan, Italy). Image scanning of the histological samples was performed using Nanozoomer and acquired using NDP.view2 Image viewing software (Hamamatsu, Shizuoka, Japan). The brown area was measured using a photoshop instrument (Photoshop, version 25.0, by Adobe, San Jose, CA, USA) that led to the selection of the brown area in figures with the same magnification and area.

### 4.3. Liver Histology

At sacrifice, livers were collected and weighed. The samples were immediately fixed with 4% (vol/vol) paraformaldehyde/PBS for 24 h at 4 °C, embedded in paraffin, sectioned and stained with H&E. Five images were captured for each section, and the areas occupied by lipid vacuoles were measured, using ImageJ software.

### 4.4. Statistical Analysis

The results are presented as mean ± standard error (SE), and a *p* value less than 0.05 was considered statistically significant. Repeated measurement analysis of variance (ANOVA) was used to evaluate weight changes at different time points. Two-way ANOVA (two types of diet and four different genotypes) was used for the other evaluations. The Tukey post hoc test was used to compare the different time point changes within each group or between specific groups. Statistical analysis was performed using SPSS 26.0 (IBM, Bologna, Italy) and Jamovi 2.4.11 (free online available https://www.jamovi.org/download.html).

## 5. Conclusions

In summary, the data indicate that LIGHT is involved in the regulation of fat tissue. *Tnfsf14*^−/−^ and DKO mice fed ND displayed the lowest weight of vWAT. In contrast, the *Tnfsf14*^−/−^ fed HFD displayed the greatest vWAT increase. Interestingly, DKO mice fed HFD showed the lowest increase in vWAT weight, suggesting that high fat intake affects fat depots simultaneously, requiring LIGHT signaling and immune cells. Under the same conditions, iWAT weight was unaffected, whereas significant changes occurred in the other conditions.

The results implicate lymphocytes expressing LIGHT forms as the initiating component of the cellular network regulating fat tissues. Expression of LIGHT is limited to activated effector cells, both adaptive and innate T cells, but not in B cells, implicating T cells in fat regulation. LIGHT signals through two receptors, LTβ Receptor and HVEM (*TNFRSF14*), both of which are implicated in fat regulation. Growing evidence suggests that both signaling receptors may be involved in fat regulation by modifying adipocyte differentiation [5,12,14,32]. The expression patterns of these receptors differ with the LTβR expressed in adipocytes and structural cells but not in lymphocyte lineages, whereas HVEM is prominently expressed in lymphocytes but also co-expressed with LTβR in subsets of epithelial and endothelial cells. The complex expression patterns of LIGHT, LTβR, and HVEM will require further studies to define the cellular pathways involved in the regulation of fat tissues.

## Figures and Tables

**Table 1:** Post hoc Tukey test for all experiments (*p*-value).

Type of Comparison					Glucose	vWAT Weight	iWAT Weight	BAT Weight	Mean Adipocyte Area in vWAT	Mean Adipocyte Number in vWAT	Mean Adipocyte Area in iWAT	Mean Adipocyte Number in iWAT	UCP1 Positivity	Steatosis Percentage in Liver
**Post hoc Genotype**	**Genotype**	**Genotype**												
	WT	*Tnfsf14* ^−/−^			0.926	0.913	<0.001	0.986	0.968	0.757	0.416	**0.010**	**<0.001**	0.151
	WT	*Rag* ^−/−^			0.762	0.796	**<0.001**	0.590	0.713	0.995	0.071	0.182	**0.028**	1.000
	WT	DKO			0.500	**<0.001**	**<0.001**	0.886	**0.009**	**0.003**	**<0.001**	0.058	0.066	**<0.001**
	*Tnfsf14* ^−/−^	*Rag* ^−/−^			0.965	0.252	0.676	0.154	0.385	0.893	**0.002**	0.321	**<0.001**	0.062
	*Tnfsf14* ^−/−^	DKO			0.727	**0.021**	**0.012**	0.52	**0.001**	**<0.001**	**<0.001**	**<0.001**	**<0.001**	**<0.001**
	*Rag* ^−/−^	DKO			0.948	**0.019**	0.215	0.905	0.076	**0.004**	**0.002**	**<0.001**	0.980	**<0.001**
**Post hoc diet type**	**Diet**	**Diet**												
	ND	HF			0.098	**0.005**	**<0.001**	0.129	**0.003**	0.304	**<0.001**	0.067	0.141	**<0.001**
**Post hoc diet X genotype**	**Genotype**	**Diet**	**Genotype**	**Diet**										
	WT	ND	WT	HFD	0.926	0.958	**0.028**	1.000	0.333	0.997	0.664	0.698	1.000	0.484
	WT	ND	*Tnfsf14* ^−/−^	ND	0.999	0.999	**0.823**	1.000	0.996	1.000	0.974	0.174	**<0.001**	0.998
	WT	ND	*Tnfsf14* ^−/−^	HFD	0.732	0.992	0.443	0.935	0.365	0.991	0.105	0.015	0.033	1.000
	WT	ND	*Rag* ^−/−^	ND	0.976	1.000	1.000	0.997	1.000	0.969	0.920	0.329	0.308	0.865
	WT	ND	*Rag* ^−/−^	HFD	0.705	0.319	1.000	0.995	1.000	0.995	0.999	0.247	0.308	0.962
	WT	ND	DKO	ND	0.646	0.998	0.253	0.996	0.359	0.067	**<0.001**	0.822	0.628	1.000
	WT	ND	DKO	HFD	0.815	1.000	1.000	1.000	1.000	0.067	0.721	0.994	0.308	**<0.001**
	WT	HFD	*Tnfsf14* ^−/−^	ND	0.987	0.531	**<0.001**	0.992	0.619	0.999	0.993	0.961	**<0.001**	0.175
	WT	HFD	*Tnfsf14* ^−/−^	HFD	1.000	1.000	0.611	0.965	1.000	0.840	0.904	0.342	**0.011**	0.257
	WT	HFD	*Rag* ^−/−^	ND	1.000	0.854	**<0.001**	0.955	0.456	1.000	0.077	0.999	0.582	0.908
	WT	HFD	*Rag* ^−/−^	HFD	1.000	0.870	**0.002**	0.938	0.446	0.875	0.248	0.998	0.582	0.861
	WT	HFD	DKO	ND	1.000	0.479	**<0.001**	0.953	**<0.001**	0.250	**<0.001**	0.083	0.882	0.211
	WT	HFD	DKO	HFD	1.000	0.661	**<0.001**	1.000	0.125	0.262	**0.049**	0.284	0.582	**<0.001**
	*Tnfsf14* ^−/−^	ND	*Tnfsf14* ^−/−^	HFD	0.834	0.695	**0.001**	0.316	0.661	0.993	0.488	0.903	0.572	1.000
	*Tnfsf14* ^−/−^	ND	*Rag* ^−/−^	ND	0.999	0.995	0.894	1.000	1.000	0.980	0.345	0.999	**<0.001**	0.384
	*Tnfsf14* ^−/−^	ND	*Rag* ^−/−^	HFD	0.807	**0.02**	0.802	1.000	1.000	0.996	0.721	0.999	**<0.001**	0.613
	*Tnfsf14* ^−/−^	ND	DKO	ND	0.730	1.000	0.901	1.000	**0.042**	0.117	**<0.001**	**0.010**	**<0.001**	0.988
	*Tnfsf14* ^−/−^	ND	DKO	HFD	0.914	1.000	0.829	0.966	0.991	0.122	0.209	**0.043**	**<0.001**	**<0.001**
	*Tnfsf14* ^−/−^	HFD	*Rag* ^−/−^	ND	0.982	0.958	0.063	0.166	0.496	0.692	**0.004**	0.547	**<0.001**	0.551
	*Tnfsf14* ^−/−^	HFD	*Rag* ^−/−^	HFD	1.000	0.578	0.101	0.139	0.486	1.000	**0.016**	0.543	**<0.001**	0.768
	*Tnfsf14* ^−/−^	HFD	DKO	ND	1.000	0.639	**<0.001**	0.237	**<0.001**	**0.019**	**<0.001**	**<0.001**	**<0.001**	0.999
	*Tnfsf14* ^−/−^	HFD	DKO	HFD	1.000	0.825	0.030	0.667	0.145	**0.018**	**0.003**	**0.003**	**<0.001**	**<0.001**
	*Rag* ^−/−^	ND	*Rag* ^−/−^	HFD	0.972	0.069	1.000	1.000	1.000	0.737	0.994	1.000	1.000	1.000
	*Rag* ^−/−^	ND	DKO	ND	0.947	0.990	0.230	1.000	0.077	0.580	**0.002**	**0.018**	0.999	0.351
	*Rag* ^−/−^	ND	DKO	HFD	0.997	1.000	1.000	0.954	1.000	0.613	0.998	0.085	1.000	**<0.001**
	*Rag* ^−/−^	HFD	DKO	ND	1.000	**0.016**	0.158	1.000	0.080	**0.023**	**<0.001**	**0.011**	0.999	0.780
	*Rag* ^−/−^	HFD	DKO	HFD	1.000	**0.025**	1.000	0.931	1.000	**0.023**	0.898	0.055	1.000	**<0.001**
	DKO	ND	DKO	HFD	0.999	1.000	0.141	0.958	0.067	1.000	**0.021**	0.996	0.999	**<0.001**

Significant results were highlighted in bold in the table.

## Data Availability

The research data will be provided following detailed request to the corresponding author.
